# Self-reported knee joint instability is related to passive mechanical stiffness in medial knee osteoarthritis

**DOI:** 10.1186/1471-2474-14-326

**Published:** 2013-11-20

**Authors:** Mark W Creaby, Tim V Wrigley, Boon-Whatt Lim, Rana S Hinman, Adam L Bryant, Kim L Bennell

**Affiliations:** 1 School of Exercise Science, Australian Catholic University, Brisbane, Queensland 4014, Australia; 2 Centre for Health, Exercise & Sports Medicine, University of Melbourne, Melbourne, Victoria 3010, Australia; 3 School of Sports, Health and Leisure, Republic Polytechnic, Singapore, Singapore

**Keywords:** Knee osteoarthritis, Passive stiffness, Instability, Varus-valgus laxity

## Abstract

**Background:**

Self-reported knee joint instability compromises function in individuals with medial knee osteoarthritis and may be related to impaired joint mechanics. The purpose of this study was to evaluate the relationship between self-reported instability and the passive varus-valgus mechanical behaviour of the medial osteoarthritis knee.

**Methods:**

Passive varus-valgus angular laxity and stiffness were assessed using a modified isokinetic dynamometer in 73 participants with medial tibiofemoral osteoarthritis. All participants self-reported the absence or presence of knee instability symptoms and the degree to which instability affected daily activity on a 6-point likert scale.

**Results:**

Forward linear regression modelling identified a significant inverse relationship between passive mid-range knee stiffness and symptoms of knee instability (r = 0.27; P < 0.05): reduced stiffness was indicative of more severe instability symptoms. Angular laxity and end-range stiffness were not related to instability symptoms (P > 0.05).

**Conclusions:**

Conceivably, a stiffer passive system may contribute toward greater joint stability during functional activities. Importantly however, net joint stiffness is influenced by both active and passive stiffness, and thus the active neuromuscular system may compensate for reduced passive stiffness in order to maintain joint stability. Future work is merited to examine the role of active stiffness in symptomatic joint stability.

## Background

Self-reported joint instability is a common complaint in individuals with knee osteoarthritis (OA) [1,2]. Knee joint instability can be defined as "the sudden loss of postural support across the knee at a time of weight bearing" [3,4]. Of import, sensations of instability can compromise an individual’s capacity to perform their daily activities [1,5] and are associated with poorer physical function [1,4,5].

Intuitively, sensations of knee joint instability (i.e. feelings of shifting, buckling or giving way of the knee) in patients with knee OA may be partly related to the mechanical stability of the joint. The provision of adequate resistance to motion (i.e. mechanical stiffness), is an important component of mechanical joint stability and is contingent upon both passive (e.g. ligaments) and active (i.e. muscle-tendon units) structures. Whilst mechanical stability in all three planes of motion is likely to influence sensations of joint stability, evidence suggests that frontal plane mechanics may be particularly important in those with medial knee OA. Some studies [6,7], but not all [8], have shown that patients with OA of the medial tibiofemoral joint demonstrate excessive varus-valgus passive laxity; there is also emerging evidence that passive mechanical stiffness is reduced with medial tibiofemoral OA [8,9]. While passive laxity, stiffness, and joint instability are not synonymous, it is possible that a lax or low-stiffness knee, when exposed to high frontal plane moments during locomotor activities (that are associated with the medial OA knee [10-12]), may experience joint instability. In contrast, patients with stiffer joint structures may report less instability given greater resistance against external perturbations. However, it is not known if knee joint passive mechanical stiffness is related to the symptomatic stability of the medial OA knee. An understanding of the relationship between self-reported knee instability and stiffness/instability may help guide the development of approaches to minimise symptomatic instability in this population.

Measurements related to frontal plane knee laxity (knee varus-valgus range-of-motion or medial joint opening on varus-valgus stress xray) indicate that such laxity does not differ between self-reported stable and unstable OA knees [2,3,13]. Similarly, frontal plane knee laxity (as indicated by medial joint opening on varus-valgus stress xray) is not related to the severity of symptomatic instability [14,15]. These previous investigations however, did not separate laxity measurements into varus and valgus, but rather measured knee laxity through the entire varus-valgus range-of-motion. Conceivably, angular laxity under varus and valgus loading may hold a different relationship with symptomatic knee instability, and this is worthy of investigation. An additional consideration is that passive knee laxity (i.e. range-of-motion), is not necessarily indicative of passive mechanical stiffness (i.e. resistance to motion). Early work in this field clearly demonstrates that the passive stiffness of the knee is dependent upon the portion of the moment-angle curve that is evaluated: typically passive stiffness is higher toward the end of range than in the mid-range [16-18]. Thus, stiffness within a given range of the moment-angle curve is not synonymous with range-of-motion across the entire moment-angle curve. Moreover, when compared with healthy knees, those with medial tibiofemoral OA demonstrate lower varus-valgus mechanical stiffness in the mid-range, but not at the end of range [8]. Thus, it may be important to evaluate the relationship between joint instability and passive stiffness in the mid- and end-range independently, as well as the relationship between instability and maximum range-of-motion (i.e. laxity). Conceivably, the mechanical behaviour of the knee close to its usual, relatively small, varus-valgus 'operating range’ (i.e. mid-range stiffness), may be particularly important for joint function and may reveal associations with joint instability.

The aim of this study therefore, was to evaluate the relationship between self-reported instability, and varus-valgus angular laxity and passive end- and mid-range stiffness, in individuals with medial knee OA. We hypothesised that greater instability would be associated with greater angular laxity and less passive stiffness.

## Methods

### Participants

Seventy-three participants were recruited for this study, and were a convenience sample of individuals recruited for a randomised controlled trial [19]. All data reported in this study were collected at baseline prior to any intervention. All participants had tibiofemoral joint OA in at least 1 knee and fulfilled the American College of Rheumatology classification criteria [20]: age >50 years, knee pain most days of the past month, and osteophytes apparent on knee radiograph. To ensure medial tibiofemoral joint OA, the following criteria were imposed: self-reported pain on the medial aspect of the knee, osteophytes in the medial tibiofemoral compartment, and medial joint space narrowing greater than lateral joint space narrowing [21]. Exclusion criteria for the randomised controlled trial from which the participants were drawn were: a history of lower limb joint replacement; knee surgery, intraarticular steroid, or hylan G-F 20 injection within the previous 6 months; systemic arthritic condition; more than 5 degrees of valgus malalignment on radiograph; were seeking or currently receiving physiotherapy for knee OA; were intending to start or currently participating in a lower limb strengthening program; or had a severe medical condition that precluded safe participation.

Ethical approval was obtained from The University of Melbourne Human Research Ethics Committee, and from the Department of Human Services Radiation Advisory Committee. Written informed consent was provided by participants at enrollment. Participants were initially screened over the telephone and those eligible underwent a standardized anteroposterior (AP) weight-bearing radiograph to ascertain knee alignment and OA severity. Participants fulfilling radiographic eligibility criteria were enrolled into the study.

### Radiographic analysis

An AP extended weight-bearing radiograph of the most painful knee was used to assess knee alignment and OA severity. When both knees were equally painful, the dominant knee was deemed the study knee. Disease severity was assessed using the Kellgren/Lawrence (K/L) scale [22], in which higher grades indicate greater severity. Anatomic knee alignment was determined using the methods of Moreland et al. [23] and was evaluated by one investigator (B-WL), with excellent intrarater reliability (intraclass correlation coefficient [ICC] 0.97 based on 10 randomly selected radiographs measured 1 week apart). Mechanical knee alignment was then predicted using the regression equation from Hinman et al. [24]. In this study, neutral alignment is reported as 180°, with lower numbers indicating more varus malalignment.

### Self-report measure of knee instability

Based on the work of others [1], the presence and severity of knee instability was self-reported using a 6-point likert scale (see Table 1) in response to the query "To what degree does giving way, buckling, or shifting of the knee affect your level of daily activity?". The test-retest reliability of this tool has previously been determined as adequate (ICC_2,1_ = 0.76) in a population including individuals with knee OA [1].

**Table 1:** Frequency of responses to knee instability questionnaire in patients with symptomatic medial compartment knee OA (n = 73)

	**Frequency**		**Cumulative**
	**n**	**%**	**%**
**To what degree does giving way, buckling or shifting of the knee affect your level of daily activity?**			
0 = The symptom prevents me from all daily activity	0	0.0	0.0
1 = The symptom affects my activity severely	10	13.7	13.7
2 = The symptom affects my activity moderately	14	19.2	32.9
3 = The symptom affects my activity slightly	20	27.4	60.3
4 = I have the symptom but it does not affect my activity	8	11.0	71.2
5 = I do not have giving way, buckling, or shifting of the knee	21	28.8	100.0

### Knee joint laxity and stiffness

**Figure 1:**
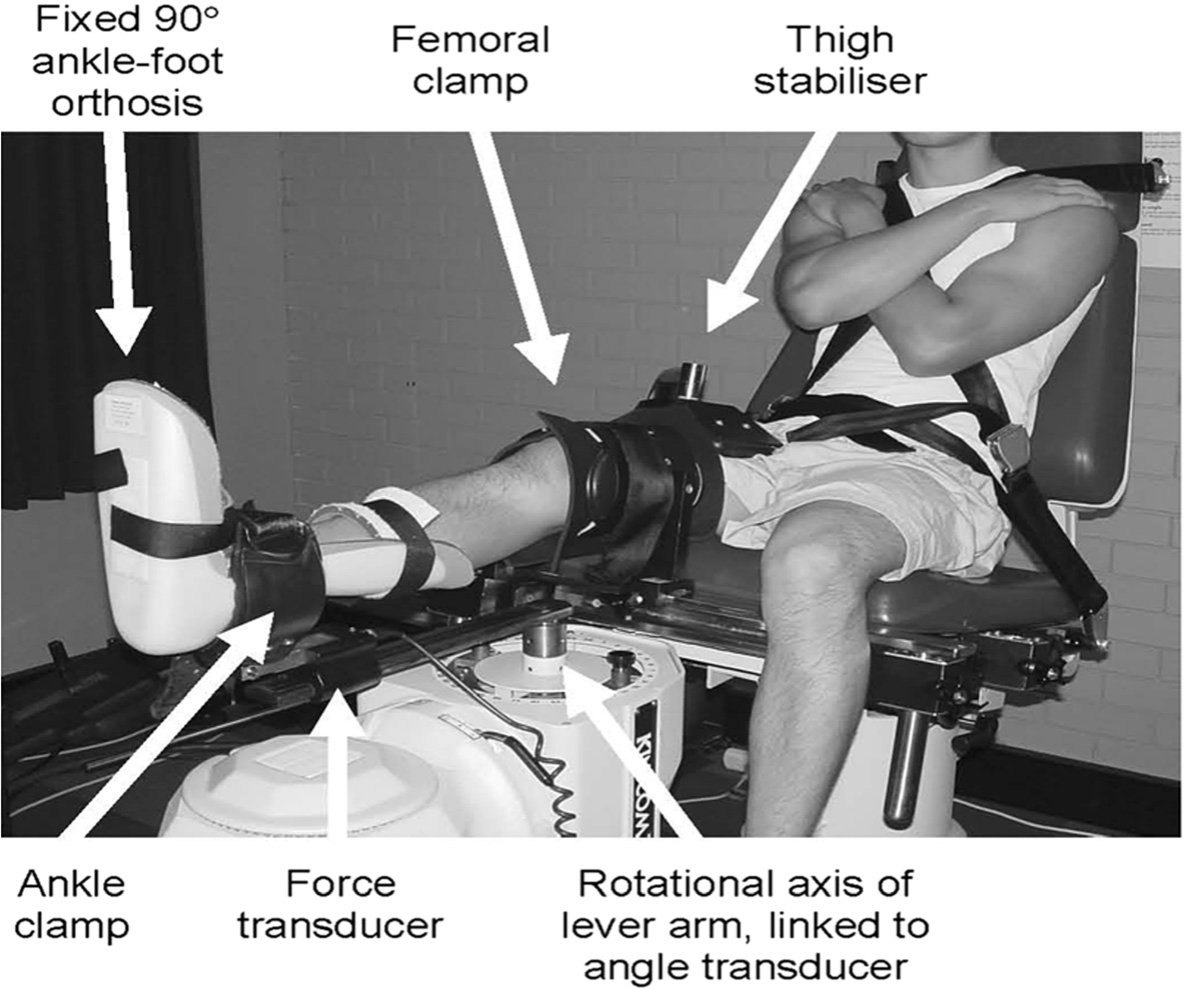
Experimental setup for the evaluation of passive knee varus-valgus laxity and stiffness.

Passive varus-valgus laxity and stiffness of the knee joint was evaluated on the same day as the questionnaire measures, using previously published techniques [8,25,26]. Briefly, participants were seated in a modified Kin-Com 125-AP dynamometer (Chattecx Corp., Chattanooga, TN, USA), with the knee secured in 20° flexion [6,8] (Figure 1). Participants wore shorts during testing, to ensure clothing did not influence the resistance to motion of the leg. The ankle was secured in 90° flexion with an ankle-foot orthosis to a load cell on the horizontal lever arm of the dynamometer, and the tibiofemoral joint directly above, and intersected by, the lever arm axis of rotation, thus ensuring a gravity-neutral position. Following a period of familiarisation to ensure the participant was comfortable with the test procedure, was not experiencing pain, or contracting the muscles crossing the knee joint [25], varus and valgus angles were determined by passive rotation to the point where 12 N.m of passive resistance was reached [8]. The leg was then passively rotated by the dynomometer from varus to valgus and valgus to varus at 5 degrees per second [8]. This movement was repeated 10 times, with the extracted data averaged across the 10 rotations.

Analog force and lever arm angle were sampled directly from the Kin-Com at 100 Hz by 16-bit analog-to-digital conversion (Micro 1401, Cambridge Electronic Design, UK). Joint torque (N.m) was computed as the product of the force (Newtons) recorded at the ankle and the lever arm length (meters; measured from the axis of rotation at the knee to the force transducer at the ankle). The neutral lever arm angle was defined at zero varus-valgus force, and on this basis, varus and valgus ranges were separated. Passive mechanical stiffness was defined as the change in joint torque divided by change in joint angle (N.m/°). End-range varus and valgus stiffness was calculated over the last 25% of the range moving in a varus and valgus direction, respectively. Mid-range stiffness was calculated from the averaged varus and valgus movement over a 2° window, 1° either side of mechanical neutral [8,9]. Test-retest reliability of the angular laxity and stiffness measures were excellent when measured a week apart in 10 people with medial tibiofemoral OA (ICC_2,1_ = 0.87 to 0.97).

### Statistical analysis

Analyses were performed using SPSS for Windows (Version 19, IBM Corporation, Armonk, NY, USA). Forward stepwise linear regression modelling was used to determine the influence of the laxity and stiffness measures upon the presence and severity of self-reported knee instability. Given that the outcome variable in these analyses (presence and severity of self-reported knee instability) is ordinal, thorough checking of the data were performed to ensure that the assumptions of parametric statistics were met. This involved the calculation of both Pearson (parametric) and Spearman (non-parametric) correlation coefficients to determine the degree of correlation between the presence and severity of self-reported knee instability and indices of knee angular laxity and stiffness. Only the explanatory variables demonstrating significant and similar correlations coefficients in both parametric and non-parametric statistics were deemed eligible for regression analyses. Further, the data were checked to ensure that the standard assumptions of linear regression were met, that is: (i) an approximately linear relationship between the explanatory variables and the outcome variables; (ii) residuals are normally distributed (Kolmogorov-Smirnov test with Lilliefors significance correction), and (iii) homoscedastic variance was present [27]. Age, gender, height and body mass were included as covariates in the regression analyses [8,28]. An *a priori* alpha level of 0.05 was set for all analyses.

## Results

Participant characteristics are shown in Table 2. Frequency of disease severity and gender was relatively equally distributed across the sample. On average, the study sample could be classified as overweight and having varus knee malalignment. Sixty percent of participants reported that symptoms of giving way, buckling, or shifting of the knee affected their daily activity at least slightly (Table 1). In 33%, the impact of instability on daily activity was moderate or greater and in 14%, it was severe. Eleven percent of participants reported symptoms of instability, but that these symptoms did not affect their daily activity. Average varus and valgus angular laxity for the entire cohort was 9.1° ± 2.8° and 8.9° ± 2.9°, respectively. Thus, total angular laxity range for the cohort was 18.0° ± 5.4°. End-range stiffness in varus and valgus was 1.62 Nm/° ± 0.46 Nm/° and 1.70 Nm/° ± 0.40 Nm/°, respectively. In the mid-range, stiffness was 1.50 Nm/° ± 0.60 Nm/°. The measures of laxity and stiffness separated according to self-reported knee instability are reported in Table 3.

**Table 2:** Summary of participant characteristics (n = 73)

	**Mean (SD)**
**Age (years)**	63.45 (8.17)
**Height (m)**	1.68 (0.09)
**Mass (kg)**	82.2 (14.5)
**BMI (kg/m**^**2**^**)**	29.3 (4.9)
**Sex (n (%))**	
Male	40 (54.8)
Female	33 (45.2)
**Disease severity **^†^**(n (%))**	
Grade 2	22 (30.1)
Grade 3	21 (28.8)
Grade 4	30 (41.1)
**Mechanical Alignment (°)**	175.7 (3.3)

^†^indicates Kellgren and Lawrence disease severity of the study limb.

**Table 3:** Mean (SD) laxity indices separated according to self-reported knee instability score

**Variable**	**Self-reported knee instability score**					
	**0 (n = 0)**	**1 (n = 10)**	**2 (n = 14)**	**3 (n = 20)**	**4 (n = 8)**	**5 (n = 21)**
Valgus laxity (°)	-	10.51 (2.90)	8.79 (1.95)	8.95 (2.70)	8.32 (2.90)	8.55 (3.63)
Varus laxity (°)	-	-10.56 (3.61)	-9.07 (2.53)	-9.00 (3.00)	-9.03 (2.02)	-8.5 (2.57)
Total laxity (°)	-	21.07 (6.14)	17.86 (4.16)	17.95 (5.45)	17.35 (4.61)	17.05 (5.87)
Valgus stiffness (Nm/°)	-	1.58 (0.40)	1.66 (0.19)	1.71 (0.37)	1.67 (0.49)	1.78 (0.50)
Varus stiffness (Nm/°)	-	1.38 (0.45)	1.54 (0.33)	1.81 (0.52)	1.56 (0.34)	1.63 (0.51)
Mid-range stiffness (Nm/°)	-	1.14 (0.41)	1.44 (0.56)	1.52 (0.58)	1.59 (0.48)	1.68 (0.69)

**Figure 2:**
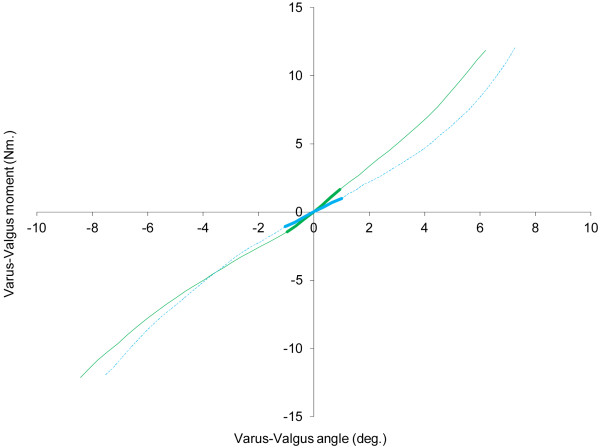
**Net moment-angle curves for two study participants are depicted.** One participant reported no symptoms of instability (solid green line), the other participant reported that instability severely affected activity (dashed blue line). Mid-range passive stiffness, as indicated by the gradient of the thickened portion of the lines, is noticeably greater in the participant with no symptoms of instability, yet total range-of-motion is similar in both participants.

Pearson and Spearman correlations (Table 4) indicate that the only laxity index significantly and similarly correlated with self-reported instability was mid-range stiffness (r = 0.27, P = 0.021; rho = 0.26, P = 0.026), whereby less mid-range stiffness was associated with instability having a greater influence upon daily activities. This finding is illustrated for two exemplar participants in Figure 2. Valgus angular laxity was correlated with self-reported instability in Spearman correlation (rho = -0.23, P = 0.048), but this was not reflected in the Pearson correlation (r = -0.17, P = 0.148).

**Table 4:** Pearson and Spearman correlation coefficients between self-reported instability and laxity indices

**Variable**	**Pearson**		**Spearman**	
	**r**	**P**	**rho**	**P**
Valgus laxity (°)	-0.17	0.148	-0.23	0.048*
Varus laxity (°)	-0.19	0.104	-0.17	0.149
Total laxity (°)	-0.19	0.102	-0.21	0.078
Valgus stiffness (Nm/°)	0.15	0.202	0.09	0.432
Varus stiffness (Nm/°)	0.12	0.306	0.13	0.272
Mid-range stiffness (Nm/°)	0.27	0.021*	0.26	0.026*

* indicates significant correlation (P < 0.05).

Mid-range stiffness was entered into stepwise regression because (i) it was significantly and similarly correlated with self-reported instability in Pearson and Spearman bivariate correlations, and (ii) the data met the standard assumptions required for valid linear regression modelling, including the normal distribution of residuals (*P* = 0.20). The other laxity indices were not significantly and consistently correlated with self-reported instability, and thus were not considered eligible for linear regression modelling. The regression model for mid-range stiffness indicates that this index is a significant independent predictor of self-reported instability, explaining 7% of the variance in this parameter (B = 0.65, SE = 0.27, P = 0.021). The nature of this relationship was such that less stiffness in the mid-range was associated with instability episodes having a greater reported influence upon daily activities.

## Discussion

Our findings are consistent with those of earlier studies that demonstrate self-reported knee instability is common and influences daily activities in a large proportion of individuals with knee OA [1,29], and medial tibiofemoral OA specifically [2,5]. Contrary to our hypothesis, self-reported instability was not correlated with varus-valgus angular laxity or end-range passive stiffness. In agreement with our hypothesis, self-reported knee instability was significantly correlated with mid-range passive stiffness; that is, less passive stiffness was associated with greater self-reported instability. These data suggest that lower passive stiffness in the mid-range is an important component of the joint instability associated with knee OA.

The relationship between self-reported instability in knee OA and varus-valgus laxity has previously been investigated using joint opening under stress radiography [2,13-15] and total measured range-of-motion in response to a fixed torque [3]. The stress radiography technique used defines "medial knee laxity" as the change in medial joint space width from extreme valgus (medial joint open) to extreme varus (medial joint closed), a movement more akin to the total varus-valgus angular laxity measure. Despite these differences in measurement technique, and different approaches to normalization for body size, findings with respect to total varus-valgus angular laxity are consistent across the literature, illustrating that angular laxity is not related to perceived joint instability [2,3,13-15]. Our data extend these findings, indicating that isolated varus and valgus angular laxity is not related to perceived joint instability. In common, these measures of angular laxity quantify the movement of the joint beyond what it is likely to typically experience during functional activities i.e. greater than 5 deg varus or valgus [30,31]. This may explain the absence of a relationship between current measures of angular laxity and joint instability.

To our knowledge, the relationship between passive knee stiffness and knee instability in OA has not previously been evaluated. Earlier work has revealed that compared with healthy knees, OA knees have less passive varus-valgus stiffness in the mid-range, and at some angles in the overall range of motion [8,9]. Extending these findings, our current study shows that only mid-range passive stiffness was related to knee instability. This provides further evidence that the mechanical behaviour of the joint in the mid-range is functionally important, and that end-range mechanics may be of less relevance to knee function. Indeed, our mid-range measurement is likely to be within the knee’s relatively small varus-valgus operating range during daily activities such as walking gait [30,31].

Knee stability is provided by the active neuromuscular system (muscle-tendon function), passive restraint (ligaments and other passive tissues), and stabilizing joint forces. In our work, we isolated the effects of passive restraint in the frontal plane; in the functional mid-range, passive restraint (stiffness) was greater in more stable knees. Conceivably, a stiffer passive system may contribute toward greater resistance to frontal plane motion during gait; this may be particularly important in medial OA knees given the high varus (i.e. external adduction) moments they are exposed to [10-12,32,33]. Mechanically, a less stiff, more compliant, system will be exposed to greater frontal plane motion, which may contribute toward both joint instability and cartilage damage [34,35].

Importantly, it is the combined effect of passive restraint, the active neuromuscular system, and joint forces that will determine joint stability during gait and other activities, and our data indicate that only a modest amount of the variance in self-reported instability (7%) is explained by passive mid-range stiffness. In light of this modest relationship and the low magnitude of absolute passive varus-valgus torques acting on the knee in the mid-range (i.e. a cohort average of 1.50 Nm/° ± 0.60 Nm/°), it is conceivable that the variance in self-reported instability explained by active neuromuscular control may be much greater than that explained by passive mid-range stiffness. To our knowledge, the frontal plane stiffness of the active neuromuscular system in OA knees has not previously been directly quantified. Some insight regarding active stiffness of the knee joint may be gleaned from our knowledge of local muscle properties. For example, recent evidence indicates that the net isometric strength of the quadriceps and hamstrings is lower in the presence of symptomatic knee instability [3], suggesting that a stronger active neuromuscular system may be associated with less symptomatic instability of the knee.

A knee with less passive mechanical stiffness will likely place a greater burden on active neuromuscular systems, which may or may not be able to compensate for the passive deficit in stiffness. Consistent with this, one electromyographically-derived muscle co-contraction index suggests that active neuromuscular control (reflective of stiffness) in the frontal plane is higher in unstable knees in response to a perturbation during walking [13], although there is evidence to the contrary in response to a perturbation when standing [14]. A low-stiffness knee surrounded by good active neuromuscular support may exhibit less symptoms of instability than one with poor active neuromuscular support. In regard to the current study’s results, such variability in available compensatory support would confound a closer relationship between purely passive stiffness and episodes of instability.

Although novel, our study has limitations. First, our study was cross-sectional in design so it is not possible to determine the cause and effect relationship between passive knee stiffness and instability. We have proposed a rationale whereby the mechanical stiffness of the knee may influence sensations of joint stability. Although unlikely, we cannot rule out the possibility that perceived stability of the knee influences the mechanical stability of the joint. Second, our findings are limited to the varus-valgus stiffness of the knee. Conceivably, sagittal and transverse plane stiffness of the knee may explain additional variance in perceived joint instability and this is worthy of investigation in future research. In the current study however, we focussed upon varus-valgus stiffness, given the high frontal plane loads and reported involvement of passive varus-valgus stiffness in medial knee OA [8,10-12]. Further, given the probable involvement of the active neuromuscular system in joint stability [3,13], and that it may moderate the relationship between passive stiffness and symptomatic instability, consideration of local muscle properties alongside measures of passive joint stiffness in future work may provide a clearer picture of the relative contribution of active and passive stiffness in symptomatic knee instability.

## Conclusions

To conclude, our data indicate that the passive mechanical stiffness of the knee is associated with self-reported symptoms of instability in people with medial knee OA. Conceivably the lower passive stiffness observed in those with symptoms of instability may contribute toward a more unstable joint environment. This may have important implications for addressing symptoms of instability in those with medial knee OA. However, these data should be interpreted with caution as compensations by the active neuromuscular system in those with reduced passive stiffness are yet to be determined.

## Competing interests

The authors declare that they have no competing interests.

## Authors’ contributions

MC participated in the conception of the study, analysis and interpretation of data, and drafted the manuscript; TW participated in the conception, design and coordination of the study, analysis and interpretation of data, and helped in the drafting and revision of the manuscript; B-WL acquired the data and participated in data analysis; RH participated in the conception, design and coordination of the study, interpretation of data, and revision of the manuscript; AB participated in the interpretation of data, and helped in the drafting and revision of the manuscript; KB participated in the conception, design and coordination of the study, interpretation of data, and revision of the manuscript. All authors read and approved the final manuscript.

## Pre-publication history

The pre-publication history for this paper can be accessed here:

http://www.biomedcentral.com/1471-2474/14/326/prepub
